# Pharmacological postconditioning with diazoxide attenuates ischemia/reperfusion-induced injury in rat liver

**DOI:** 10.3892/etm.2013.941

**Published:** 2013-01-31

**Authors:** Y.S. TIAN, T.Z. RONG, Y.L. HONG, L. MIN, P.G. JIAN

**Affiliations:** 1 Chongqing Key Laboratory of Hepatobiliary Surgery, Second Affiliated Hospital, Chongqing Medical University, Chongqing, P.R. China; 2 Department of Hepatobiliary Surgery, Second Affiliated Hospital, Chongqing Medical University, Chongqing, P.R. China

**Keywords:** diazoxide, ischemic postconditioning, reperfusion injury, liver

## Abstract

It has been demonstrated that ischemic postconditioning (IPO) is capable of attenuating ischemia/reperfusion (I/R) injury in the heart. However, the novel role of pharmacological postconditioning in the liver remains unclear. In this study, the hypothesis that diazoxide postconditioning reduces I/R-induced injury in rat liver was tested. Rats were assigned randomly to the sham-operated control, I/R (occlusion of the porta hepatis for 60 min, followed by a persistent reperfusion for 120 min), diazoxide ischemic postconditioning (DIPO; occlusion of the porta hepatis for 60 min, then treatment with diazoxide for 10 min reperfusion, followed by a persistent reperfusion for 110 min) or 5-hydroxydecanoate (5-HD)+DIPO (occlusion of the porta hepatis for 60 min, then treatment with diazoxide and 5-HD for 10 min reperfusion, followed by a persistent reperfusion for 110 min) groups. The alanine aminotransferase (ALT) and aspartate transaminase (AST) levels were assayed. The expression levels of protein kinase c-ε (pkc-ε), cytochrome c (cyt-c), caspase-3 and bcl-2 protein were determined by western blotting. The serum levels of ALT and AST and expression levels of cyt-c and caspase-3 were significantly lower in the DIPO group (P<0.05). However, the protein expression levels of pkc-ε and bcl-2 were markedly increased in the DIPO group (P<0.05). 5-HD abrogated the protective effects of DIPO. The data of the present study provide the first evidence that DIPO protects the liver from I/R injury by opening the mitochondrial K_ATP_ channels, activating and upregulating pkc-ε and inhibiting the activation of the apoptotic pathway by decreasing the release of cyt-c and the expression of caspase-3 and increasing bcl-2 expression.

## Introduction

Prolonged ischemia may lead to irreversible tissue injury. Reperfusion may also cause tissue injury and the composite damage is known as ischemia-reperfusion (I/R) injury. Currently the measures taken to protect the organs and tissues against I/R injury are mainly ischemic preconditioning (IPC) and ischemic postconditioning (IPO). In the protection of organs and tissues from I/R injury, IPC and IPO have similar functions. It has been reported that IPC (1–5) and IPO (6–10) have protective effects on the myocardium, including the reduction of infarct size, improvement of coronary blood flow and myocardial reperfusion. This is achieved by activating the prosurvival kinases PI3K-Akt, eNOS, NO and p70S6K, A adenosine receptors and protein kinases, including Akt and Erk1/2, guanylate cyclase, cGMP-dependent protein kinase (protein kinase G, PKG) and protein kinase c (pkc) which results in pkc-ε opening the ATP-dependent mitochondrial potassium (mito-K_ATP_) channels (11). This inhibits the opening of the mitochondrial permeability transition pore (mPTP) (12). Pharmacological preconditioning and pharmacological postconditioning have functional similarities to the associated phenomena, as well as IPC and IPO. It has been reported that sevoflurane preconditioning improves ventricular function and recovery from myocardial stunning and sevoflurane postconditioning reduces reperfusion arrhythmias without affecting the severity of myocardial stunning (13). However, the effects of diazoxide (a selective mito-K_ATP_ channel opener) postconditioning in the liver with ischemic reperfusion injury remain unclear.

In the present study, to clarify these issues, the effects of diazoxide postconditioning on I/R-induced injury in rat liver were investigated.

## Materials and methods

### Diazoxide and 5-hydroxydecanoate (5-HD)

Diazoxide (a selective mito-K_ATP_ channel opener) and 5-HD (a selective mito-K_ATP_ channel inhibitor) were purchased from Sigma (St. Louis, MO, USA). Diazoxide was dissolved and diluted with a solution of sodium hydroxide (0.1 mol/l) while 5-HD was dissolved and diluted with distilled water.

### Experimental groups and protocols

**Figure 1:**
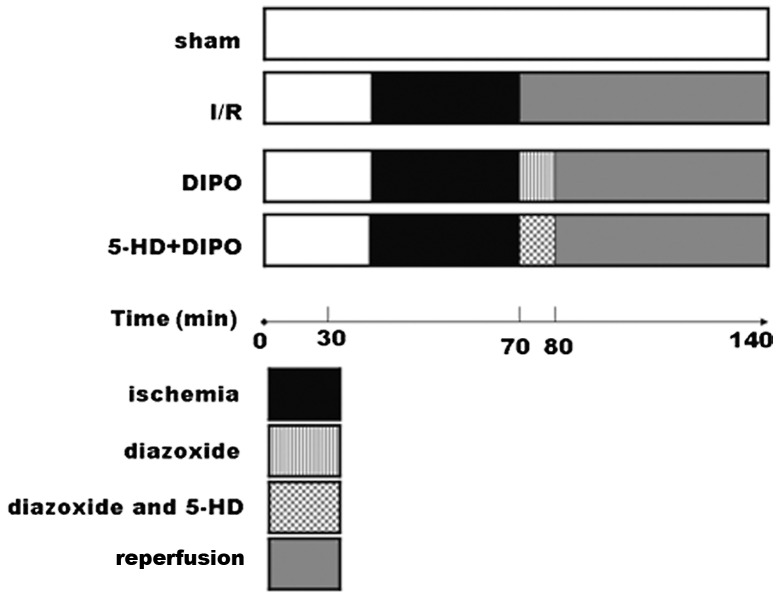
Experimental groups and protocols. I/R, ischemia/reperfusion; DIPO, diazoxide ischemic preconditioning; 5-HD, 5-hydroxydecanoate.

Adult male Sprague-Dawley rats (weight, 200–250 g) were used as the experimental animals. The rats were kept in a temperature-controlled environment (25 to 30°C) and provided with a standard diet with water *ad libitum*. Four groups were studied (n=7/group): the sham-operated control group; I/R group (occlusion of the porta hepatis for 60 min, followed by a persistent reperfusion for 120 min); DIPO (diazoxide ischemic preconditioning) group [occlusion of the porta hepatis for 60 min, then treatment with diazoxide (30 *μ*mol/l) for 10 min, followed by a persistent reperfusion for 120 min], 5-HD+DIPO group [occlusion of the porta hepatis for 60 min, then treatment with diazoxide (30 *μ*mol/l) and 5-HD (300 *μ*mol/l) for 10 min, followed by a persistent reperfusion for 110 min]. After a midline laparatomy incision, an atraumatic vascular clip was placed on the vessels, blocking the portal venous and hepatic arterial blood supply to the median and left lateral lobes of the liver and resulting in ∼70% mouse liver I/R injury. After 60 min ischemia, the diazoxide or diazoxide and 5-HD was injected through the tail vein for 10 min to keep pace with the reperfusion. The sham-operated animals underwent the same surgical procedure as the other animals with the exception that the vessel clips were not applied. Blood samples and liver tissues from each group were obtained for analysis after reperfusion for 120 min (Fig. 1).

### Serum liver function assay

Blood samples were obtained after reperfusion for 120 min. Serum alanine aminotransferase (ALT) and aspartate transaminase (AST) levels were measured with a standard clinical automated analyzer (ILab 600, Instrumentation Laboratory, Shimadzu Co., Kyoto, Japan).

### Protein expression levels of pkc-ε, cytochrome-c (cyt-c), caspase-3 and bcl-2

The animal proteins were extracted from hepatic tissues and quantified with the Bradford assay. Equal amounts of protein (50 *μ*g) were separated by sodium dodecyl sulfate-polyacrylamide gel electrophoresis (SDS-PAGE). These proteins were transferred onto polyvinylidene difluoride (PVDF) membranes. The membranes were incubated overnight at 4°C with rabbit polyclonal anti-pkc-ε (diluted 1:500), rabbit polyclonal anti-cyt-c (diluted 1:500), rabbit polyclonal anti-caspase-3 (diluted 1:500) and rabbit polyclonal anti-bcl-2 (diluted 1:500) separately, followed by the horseradish peroxidase-labeled secondary antibody (diluted 1:2,000, Santa Cruz Biotechnology Inc., Santa Cruz, CA, USA). The membranes were re-incubated with β-actin (β-actin; diluted 1:5,000, Santa Cruz Biotechnology Inc.) as a control for protein loading. The detection procedures were performed using an ECL advance western blotting detection kit, in a GeneGnome system (Synoptics, Cambridge, UK). Band intensity volumes were measured using Quantity One software (Bio-Rad, Hemel Hempstead, UK).

### Statistical analysis

All data are presented as the mean ± standard deviation (SD). Data were analyzed using ANOVA for multiple comparisons. Comparisons between two groups were performed using a t-test. All analyses were performed with the SPSS software (version 18.0, SPSS Inc., Chicago, IL, USA). P<0.05 was considered to indicate a statistically significant difference.

## Results

### Physiological function of DIPO in hepatic I/R injury

**Figure 2:**
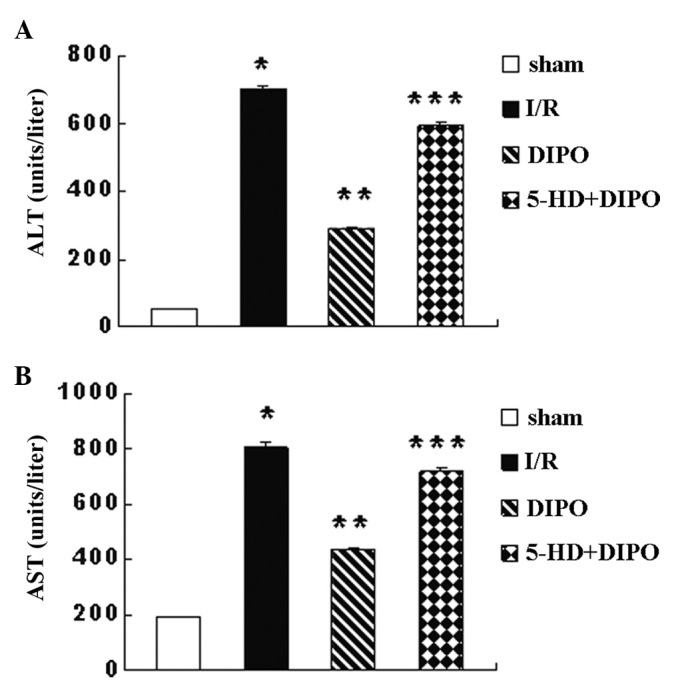
ALT and AST levels. After 60 min of ischemia and 2 h of reperfusion, serum levels of (A) ALT and (B) AST were determined. Compared with the sham group, the ALT and AST levels in the I/R group were significantly increased. Compared with the I/R group, the ALT and AST levels in the DIPO group were significantly decreased. However, 5-HD eliminated the protective effect of DIPO. Compared with the DIPO group, the ALT and AST levels in the 5-HD+DIPO group were significantly increased. ^*^P<0.05 vs. sham; ^**^P<0.05 vs. I/R; ^***^P<0.05 vs. DIPO (n=7). ALT, alanine aminotransferase; AST, aspartate transaminase; I/R, ischemia/reperfusion; DIPO, diazoxide ischemic postconditioning; 5-HD, 5-hydroxydecanoate.

Reperfusion for 10 min with diazoxide, following immediately after 60 min ischemia of the left liver lobes was applied to the DIPO group to determine if DIPO was able to attenuate I/R injury. Serum levels of ALT and AST (Fig. 2) were measured after 2 h of reperfusion following 60 min of ischemia and were significantly different among the groups. The ALT and AST levels in the I/R group were significantly higher than those in the sham-operated control mice. DIPO treatment significantly reduced the serum levels of ALT and AST compared with those in the I/R group (Fig. 1). However, 5-HD may abrogate the protective effect of DIPO (Fig. 2).

### Protein expression levels of pkc-ε, cyt-c, caspase-3 and bcl-2

**Figure 3:**
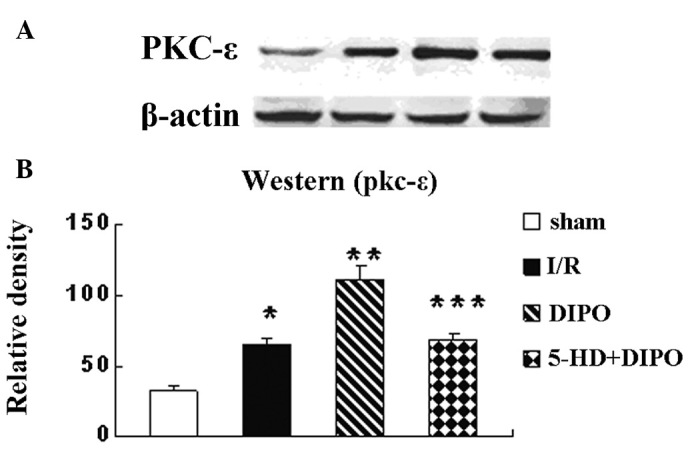
Expression levels of pkc-ε by western blotting. After 60 min of ischemia and 2 h of reperfusion, expression levels of pkc-ε in DIPO-treated hepatic tissues were determined by western blot analysis. Treatment with diazoxide (30 *μ*mol/l) activated the pkc-ε protein. However, 5-HD (300 *μ*mol/l) significantly abrogated the expression levels of pkc-ε. β-actin was used as an equal loading control. (A) The corresponding mean optical densities in each group are shown. (B) Bar graph shows the ratio of pkc-ε to β-actin. All values are means ± SD; n=7. ^*^P<0.05 vs. sham; ^**^P<0.05 vs. I/R; ^***^P<0.05 vs. DIPO. DIPO, diazoxide ischemic postconditioning; 5-HD, 5-hydroxydecanoate; I/R, ischemia-reperfusion; pkc-ε, protein kinase c-ε.

**Figure 4:**
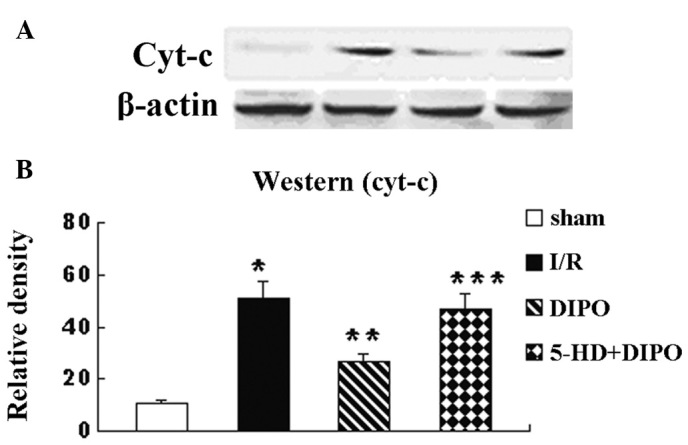
Western blot analysis of cyt-c. After 60 min of ischemia and 2 h of reperfusion, expression levels of cyt-c in DIPO-treated hepatic tissues were determined by western blot analysis. I/R treatment activated the rat liver apoptotic pathway. Diazoxide (30 *μ*mol/l) postconditioning inhibited the release of cyt-c from the mitochondrion to the cytoplasm. However, 5-HD (300 *μ*mol/l) significantly abrogated the effect of diazoxide postconditioning. β-actin was used as an equal loading control. (A) The corresponding mean optical densities in each group are shown. (B) Bar graph shows the ratio of cyt-c to β-actin. All values are means ± SD; n=7. ^*^P<0.05 vs. sham; ^**^P<0.05 vs. I/R; ^***^P<0.05 vs. DIPO. DIPO, diazoxide ischemic postconditioning; 5-HD, 5-hydroxydecanoate; I/R, ischemia-reperfusion; cyt-c, cytochrome c.

**Figure 5:**
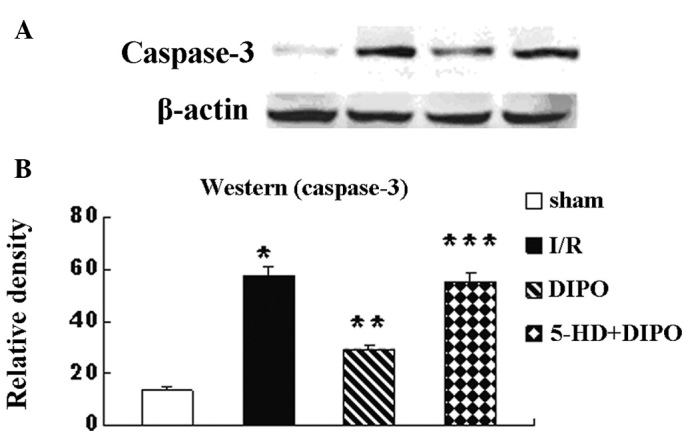
Western blot analysis of caspase-3 expression. After 60 min of ischemia and 2 h of reperfusion, expression levels of caspase-3 in DIPO-treated hepatic tissues were determined by western blot analysis. I/R treatment activated the rat liver apoptotic pathway which led to an increase in caspase-3 protein expression levels. Diazoxide (30 *μ*mol/l) postconditioning inhibited the increases of caspase-3. However, 5-HD (300 *μ*mol/l) significantly abrogated the effect of diazoxide postconditioning. β-actin was used as an equal loading control. (A) The corresponding mean optical densities in each group are shown. (B) Bar graph shows the ratio of caspase-3 to β-actin. All values are means ± SD; n=7. ^*^P<0.05 vs. sham; ^**^P<0.05 vs. I/R; ^***^P<0.05 vs. DIPO. DIPO, diazoxide ischemic postconditioning; 5-HD, 5-hydroxydecanoate; I/R, ischemia-reperfusion.

**Figure 6:**
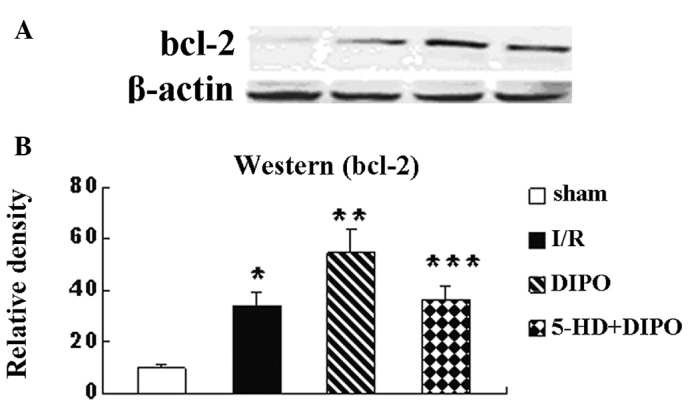
Western blot analysis of bcl-2 expression. After 60 min of ischemia and 2 h of reperfusion, expression levels of bcl-2 in DIPO-treated hepatic tissues were determined by western blot analysis. Diazoxide (30 *μ*mol/l) postconditioning activated the antiapoptotic pathway which led to significant increases in bcl-2 expression levels. Treatment with 5-HD (300 *μ*mol/l) significantly abrogated the effect of diazoxide postconditioning. β-actin was used as an equal loading control. (A) The corresponding mean optical densities in each group are shown. (B) Bar graph shows the ratio of bcl-2 to β-actin. All values are means ± SD; n=7. ^*^P<0.05 vs. sham; ^**^P<0.05 vs. I/R; ^***^P<0.05 vs. DIPO. DIPO, diazoxide ischemic postconditioning; 5-HD, 5-hydroxydecanoate; I/R, ischemia-reperfusion.

To assess how DIPO protects the liver from I/R injury, the protein expression levels of pkc-ε were measured by western blot analysis. The results revealed that the expression levels of pkc-ε in the liver tissues were significantly increased in the I/R group compared with those in the sham group, were significantly higher in the DIPO-treated mice than in the I/R group and 5-HD markedly abrogated the DIPO-induced increases in pkc-ε expression (Fig. 3). It has been previously reported that the apoptosis signaling pathway is involved in the protective effect of IPO (14,15). Whether DIPO alters the activation of the liver I/R-induced apoptosis signaling pathway was also investigated. The expression levels of cyt-c, caspase-3 and bcl-2 were recorded (Figs. 4–6). The expression levels of cyt-c, caspase-3 and bcl-2 in the I/R group were significantly increased compared with those in the sham group. DIPO significantly increased the expression levels of bcl-2 and decreased the expression levels of cyt-c and caspase-3 compared with those in the I/R group. Treatment with 5-HD markedly abrogated the DIPO-induced increases in Bcl-2 expression and decreases in cyt-c and caspase-3 expression.

## Discussion

The present study demonstrated for the first time in live rat livers that DIPO protected the liver against I/R injury by activating pkc-ε and opening mito-K_ATP_ channels. The results indicated that DIPO significantly improved the function of the liver. It improved the crucial indices of ALT and AST, which reflect the function of liver, and may inhibit the apoptosis of liver cells by inhibiting the apoptotic pathway. When 5-HD, the mito-K_ATP_ channel blocker, was administered following the DIPO surgery, it was revealed that the DIPO induced protection was abrogated. Therefore, we suggest that mito-K_ATP_ is crucial in I/R injury of the rat liver.

Previous studies have demonstrated that the opening of mito-K_ATP_ channels may be involved in the cardioprotective effects of IPC and IPO (16,17). That the mito-K_ATP_ channel is the receptor responsible for the cardioprotective actions of K_ATP_ channel openers suggested that the mitochondrial level was significant in aspects of the protective effect (18). Another study demonstrated that mitochondrial protection by diazoxide preconditioning reduced the permeability of the mitochondria to exogenous cyt-c and maintained low outer membrane permeability to nucleotides. It was also revealed that diazoxide prevented increases in the permeability of the outer membrane to nucleotides and cyt-c (19). The release of cyt-c may activate the apoptotic pathway as an upstream event of caspase activation (20). It has also been demonstrated that preventing the activation of the mitochondrial apoptotic pathway may inhibit apoptosis (15) and the participation of the mitochondrial pathway has been demonstrated by the release of cyt-c from mitochondria into cytoplasmic fractions and caspase-9 cleavage (21). Caspase-9 activates caspase-3 (15) which may cause apoptosis. In that case, inhibiting the apoptosis by blocking cyt-c release or caspase activation may be a therapeutic target. An increasing number of studies have shown that bcl-2 family members, particularly bcl-2, may inhibit apoptosis by blocking cytochrome c release and inhibiting caspase-3 and -9 activation but not that of caspase-12 (14,22). The present study demonstrated that cyt-c release and active caspase-3 together indicated the activation of the caspase-dependent pathway of apoptosis and bcl-2 overexpression in the DIPO group may inhibit this pathway which was consistent with the previously mentioned findings.

The pkc family of signaling proteins, in particular pkc-δ and pkc-ε, is commonly associated with the modulation of I/R injury. Pkc-δ has been implicated as a key signaling element in the cerebral and myocardial reperfusion injury processes (23–26). Pkc-δ is associated with increased superoxide anion generation and the enhanced release of pro-apoptotic factors and cyt-c (27,28). Activation and trans-location of pkc-ε have been revealed to be crucial in triggering the cardioprotective effects of IPC and IPO (26,29–37). It has been demonstrated that postconditioning decreased the infarct size and was dependent on pkc signaling. Postconditioning was associated with significantly higher pkc-ε levels in areas of the myocardium at risk and selective isoform inhibition prevented the infarct size reduction (26). In addition, pkc-ε activity, possibly via oxygen radicals originating from the mitochondrion, is necessary for the opening of mito-K_ATP_ channels which may protect against reperfusion injury (5,32,38,39). In summary, postconditioning may promote the activation and translocation of pkc-ε and limit the reperfusion-induced pkc-δ translocation to mitochondria. In the present study it was identified that, compared with the I/R group, the expression levels of pkc-ε in the DIPO group were significantly increased and these increases were abrogated by 5-HD. It is possible that the protection the liver against I/R injury was through the activation of pkc-ε which facilitated the opening of mito-K_ATP_ channels.

The strategy of postconditioning with diazoxide is relatively simple to perform, particularly during liver transplantation, and may have the potential to be used in clinical surgery where it may improve the survival rate of patients.

In summary, the findings of the present study indicate that DIPO protects the liver from I/R injury by reducing the serum levels of ALT and AST and opening mito-K_ATP_ channels, activating and upregulating pkc-ε and inhibiting the activation of the apoptotic pathway by decreasing the release of cyt-c and the expression of caspase-3 and increasing the expression of bcl-2.
